# Evaluation of a Commercial Multiplex Real-Time PCR with Melting Curve Analysis for the Detection of *Mycobacterium tuberculosis* Complex and Five Nontuberculous Mycobacterial Species

**DOI:** 10.3390/microorganisms13010026

**Published:** 2024-12-26

**Authors:** Keun Ju Kim, Yunhee Chang, Seung Gyu Yun, Myung-Hyun Nam, Yunjung Cho

**Affiliations:** 1 Department of Laboratory Medicine, Korea University Anam Hospital, Korea University College of Medicine, Seoul 02841, Republic of Korea; koryun@korea.ac.kr (S.G.Y.); yuret@korea.ac.kr (M.-H.N.); eqcho1ku@korea.ac.kr (Y.C.); 2 Department of Biomedical Laboratory Science, Kyungnam College of Information & Technology, Busan 47011, Republic of Korea; yhchang@eagle.kit.ac.kr

**Keywords:** tuberculosis, melting curve analysis, NTM differentiation, NeoPlex TB/NTM

## Abstract

Background: Accurate and timely diagnosis of mycobacterial infections, including *Mycobacterium tuberculosis* complex (MTBC) and nontuberculous mycobacteria (NTM), is crucial for effective disease management. Methods: This study evaluated the performance of the NeoPlex TB/NTM-5 Detection Kit (NeoPlex assay, Seongnam, Republic of Korea), a multiplex real-time PCR assay that incorporates melting curve analysis, compared with the line-probe assay (LPA). The NeoPlex assay could simultaneously detect and differentiate MTBC from five other NTM species: *Mycobacterium intracellulare*, *Mycobacterium avium*, *Mycobacterium kansasii*, *Mycobacterium abscessus*, and *Mycobacterium massiliense*. A total of 91 acid-fast bacillus culture-positive samples, comprising 36 MTBC and 55 NTM isolates, were collected from the Korea University Anam Hospital. Results: The NeoPlex assay successfully detected nucleic acids in 87 of the 91 isolates (95.6%). Notably, it identified additional mycobacterial nucleic acids not detected by the LPA in eight isolates. These findings were confirmed via DNA sequencing. The assay had 100% sensitivity and specificity for *M. intracellulare*, *M. abscessus*, *M. massilense*, NTM, and MTBC, whereas it had 100% specificity and sensitivity of 90.9% and 75.0% for *M. avium* and *M. kansasii*, respectively. Conclusions: These results highlight the potential of the NeoPlex assay to enhance rapid and accurate diagnosis of mycobacterial infections, particularly in settings in which prompt treatment initiation is essential.

## 1. Introduction

Tuberculosis (TB) continues to be a major public health concern and is the leading infectious disease cause of death. The diagnosis of nontuberculous mycobacteria (NTM) infections has also been rising worldwide, likely due to advancements in diagnostics and awareness of the disease [1,2]. Mycobacteria display a range of virulence and antibiotic susceptibility. Therefore, the treatment strategy is determined by the specific species identified. Rapidly distinguishing NTM from *Mycobacterium tuberculosis* complex (MTBC) and identifying NTM at the species level is therefore essential to provide the appropriate treatment [3,4,5].

Although prevalence varies widely by geographic region, sex, and age, *Mycobacterium avium* complex species are the most common cause of NTM infections globally [6,7]. The main *M. avium* complex species are *Mycobacterium intracellulare* and *M. avium*. In contrast to TB, pulmonary diseases caused by *M. avium* complex are difficult to cure [4]. *Mycobacterium abscessus* complex species are considered the most antibiotic-resistant mycobacteria [8]. *M. abscessus* complex species mainly comprises *M. abscessus* and *Mycobacterium massiliense. M. abscessus* complex species are the second most frequent NTM species associated with lung disease [7,9]. Although *M. abscessus* complex can infect individuals with or without underlying risk factors, it is most often seen in immunosuppressed individuals or individuals with preexisting lung conditions [9]. *Mycobacterium kansasii* is the sixth most common NTM species isolated from clinical samples [10,11] and has been identified as the leading cause of pulmonary NTM disease in sub-Saharan Africa [12] and the Basque Country in northern Spain [13].

Conventional biochemical methods for identifying mycobacteria have been largely replaced by advanced technologies, such as DNA sequencing, the line-probe assay (LPA), and matrix-assisted laser desorption/ionization time-of-flight (MALDI-TOF) mass spectrometry [14]. Gene sequencing is the gold standard for NTM identification, but its use is restricted to a limited number of laboratories because of high costs, the need for specialized staff, and long processing times [15]. LPA is the assay most frequently used to identify NTM in clinical microbiology laboratories. This genotyping method uses nucleotide probes fixed to nitrocellulose strips for hybridization with specific DNA sequences. Although LPA is a reliable method, it is costly, requires specialized training, and typically has an extended turnaround time [16]. MALDI-TOF mass spectrometry has recently emerged as a promising technique, which reduces the turnaround time and is capable of identifying more than 180 species, depending on the database. However, its application in mycobacterial identification remains limited owing to challenges with protein extraction, which is complicated by the thick cell wall of mycobacteria [16,17].

In this study, we evaluated the performance of the NeoPlex TB/NTM-5 Detection Kit (NeoPlex assay; GeneMatrix, Seongnam, Republic of Korea), a multiplex real-time PCR assay that incorporates melting curve analysis. The NeoPlex assay is designed to detect MTBC and NTM, differentiating between five NTM species: *M. intracellulare*, *M. avium*, *M. kansasii*, *M. abscessus*, and *M. massiliense*. Its performance was compared with the reference test conducted at the Korean Institute of Tuberculosis, which utilizes a composite of LPA, DNA sequencing, and PCR.

## 2. Materials and Methods

### 2.1. Clinical Samples

This study was performed at Korea University Anam Hospital between July and October 2024. In total, 91 acid-fast bacillus (AFB) culture-positive samples were used, 36 samples of MTBC and 55 samples of NTM species (Table 1).

### 2.2. Standard of Care Testing for Mycobacterial Culture and Identification

Decontaminated clinical specimens were inoculated into BACTEC 960 mycobacterial growth indicator tubes (MGITs) (Becton Dickinson, Franklin Lakes, NJ, USA) and 2% Ogawa medium (Shinyang, Seoul, Republic of Korea) for mycobacterial culture. Liquid cultures were maintained for 6 weeks, whereas solid cultures were observed for 8 weeks. In positive cultures, the presence of AFB was confirmed using Ziehl-Neelsen staining. Differentiation between MTBC and NTM was conducted using the MPT 64 antigen test (SD Bioline Kit; Standard Diagnostics, Yongin, Republic of Korea) and assessment of colony morphology. NTM-positive isolates were sent to the Korean Institute of Tuberculosis (KIT), a World Health Organization (WHO) supranational TB reference laboratory, for species identification. NTM species identification was performed using the AdvanSure Mycobacteria GenoBlot assay (Invitros, Seoul, Republic of Korea), which can identify MTBC and 20 different NTM species [18]. This reverse LPA can detect multiple NTM species in a single specimen. For species that were not distinguished by the assay, further analysis was performed using multigene sequence typing. Sequencing of the 16S rRNA, *rpoB*, and *hsp65* genes was performed according to the CLSI guidelines [19]. Additionally, the ERM-plus real-time PCR kit (Invitros; not commercially available) was used to differentiate *M. abscessus* and *M. massiliense*.

### 2.3. NeoPlex Assay

Ninety-one positive cultures (MGIT or Ogawa) of previously identified mycobacterial species were analyzed using the NeoPlex assay. For cultures grown in MGIT, 0.5 mL of the culture medium was transferred to a 2 mL Eppendorf tube and gently vortexed. For cultures grown on Ogawa agar, colonies were collected and suspended in 1 mL of phosphate-buffered saline (PBS) using a 1 μL loop. DNA was extracted using the AdvanSure E3 Automated Nucleic Acid Extraction System/Nucleic Acid Detection Kit (Invitros). Real-time PCR was then performed using the CFX96 PCR system (Bio-Rad, Hercules, CA, USA) with the NeoPlex TB/NTM-5 Detection Kit, which uses a single tube containing all the necessary specific primers. These primers targeted IS*6110* (for MTBC), 16S rRNA (for *Mycobacterium* species), and proprietary markers for the five NTM species (Table 2). After amplification, melting curve analysis was performed using various dyes (Table 2). Each PCR reaction consisted of 20 μL total volume (5 μL template DNA, 5 μL TB/NTM-5 PPM, 5 μL 4X NeoPlex PCR Master Mix, and 5 μL DNase-free water). The human endogenous internal control gene *UBB* was used to monitor potential PCR inhibition. The thermocycling protocol started with a 4 min step at 50 °C, followed by a 15 min step at 95 °C, and 40 cycles of 5 s at 95 °C, 1 min at 66 °C, and 5 s at 78 °C. After the final cycle, the melting curve was obtained by gradually heating from 55 °C to 80 °C. Representative melting curve plots and temperatures are shown in Figure S1. Cycle threshold (Ct) values were only available for the internal control, IS*6110*, and 16S rRNA, whereas specific melting curve analysis was conducted to identify the five NTM species. The results were interpreted according to the manufacturer’s instructions (Table S1). This assay is not designed to detect molecular antimicrobial resistance patterns associated with MTBC or NTM.

### 2.4. Comparison of NeoPlex Assay to Standard of Care Testing

Concordance was evaluated by comparing the NeoPlex results with those of the standard of care (SOC), which served as the reference method. When NeoPlex identified the nucleic acids of the mycobacterial species detected by SOC, the results were deemed correct, regardless of any additional findings from the NeoPlex assay. If the NeoPlex assay detected additional mycobacterial signals, the extracted DNA was sent to GeneMatrix Corporation (Seongnam, Republic of Korea) for sequencing, using the same primer targets as those used in the melting curve analysis. If the NeoPlex assay did not achieve species-level identification of NTM, DNA sequencing was performed using 16S rRNA and *rpoB* primers. Sensitivity and specificity analyses were conducted for each target of the NeoPlex kit using GraphPad Prism version 10.3.1 for Windows (GraphPad Software, Boston, MA, USA). For samples in which sequencing was performed, the sequencing results were considered the reference for determining sensitivity and specificity.

## 3. Results

### 3.1. Species Identification Using NeoPlex Assay

**Figure 1:**
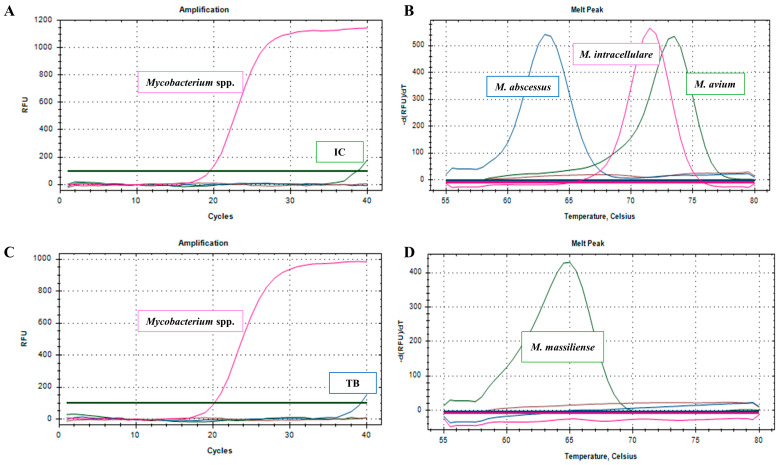
Amplification and melting curve analysis of the NeoPlex TB/NTM-5 assay. The amplification plot of the NeoPlex TB/NTM-5 assay is displayed for (**A**) a specimen (study number 2) containing *M. abscessus* and (**B**) the corresponding melting curve analysis. (**B**) The subsequent melting curve analysis reveals the presence of *M. intracellulare*, *M. avium*, and *M. abscessus*. Similarly, (**C**) shows the amplification plot for a specimen (study number 8) containing *M. massiliense.* (**D**) The subsequent melting curve analysis identified the presence of *M. massiliense*. The TB signal was amplified in the amplification plot (**C**). dRFU, order derivative relative fluorescence unit; dT, time derivative; IC, internal control; TB, tuberculosis.

The NeoPlex assay results for each run are shown in Table S2. The NeoPlex assay accurately identified and distinguished 36 MTBC and 55 NTM isolates. For species-level identification, 79 of the 91 isolates (86.8%) produced the expected results (Table 3). For eight isolates, the NeoPlex assay detected additional mycobacterial nucleic acids that were not identified by the LPA, along with the anticipated signals (Table 3, Figure 1). Two multispecies isolates (one with *M. intracellulare* and *M. avium* and the other with *M. avium* and *M. kansasii*) were only partially detected by the assay as *M. intracellulare* and *M. avium*, respectively. Additionally, one isolate of *M. avium* and one isolate of *M. kansasii* were identified as NTM, and the expected melting curve signal was not detected. Overall, the NeoPlex assay correctly identified 87 of 91 isolates (95.6%), with eight isolates (8.8%) showing signs of additional mycobacterial species. Table 3 summarizes the differences between the NeoPlex assay results and those of the SOC. Notably, a previously unidentified MTBC was identified in a specimen infected with *M. massiliense* (study number 8) during the amplification phase using the IS*6110* target (Table 4 and Figure 1C).

### 3.2. Further Analysis of Additional and Misidentification

**Figure 2:**
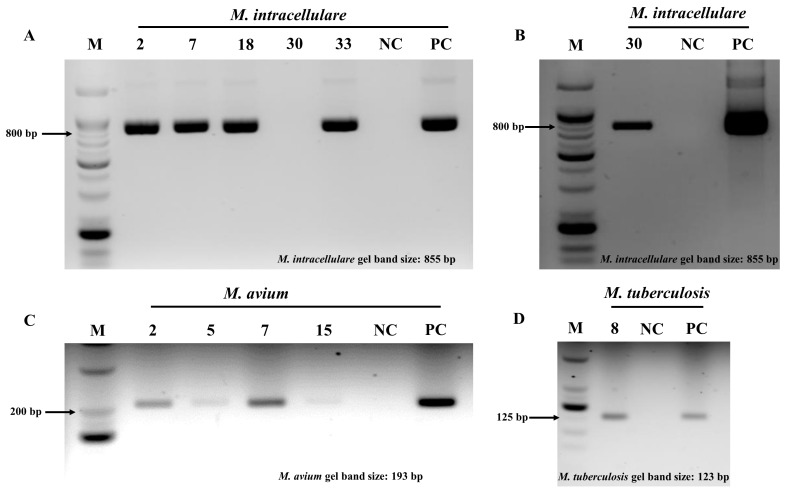
Amplification of additional signals detected using the NeoPlex TB/NTM-5 assay. (**A**) *M. intracellulare* signals were confirmed via PCR using *M. intracellulare*-specific primers. The expected size of the target is 855 bp. (**B**) A second PCR was performed for specimen number 30 using the same *M. intracellulare*-specific primers and the product of the first PCR (**A**). (**C**) *M. avium* targets were amplified via PCR using *M. avium*-specific primers. The expected size of the target is 193 bp. (**D**) MTBC target was amplified via PCR using MTBC-specific primers. The expected size of the target is 123 bp. M, DNA sizer markers; NC, negative control; PC, positive control. The numbers at the top of each column are the study numbers. The identities of the species by study number were as follows: study number 2—specimen with *M. abscessus* and additional signals of *M. intracellulare* and *M. avium*; study number 5—specimen with *M. abscessus* and additional signal of *M. avium*; study number 7—specimen with *M. abscessus* and additional signals of *M. intracellulare* and *M. avium*; study number 8—specimen with *M. massiliense* and additional signal of MTBC; study number 15—specimen with *M. intracellulare* and additional signal of *M. avium*; study number 18—specimen with *M. avium* and additional signal of *M. intracellulare*; study number 30—specimen with *M. kansasii* and additional signal of *M. intracellulare*; study number 33—specimen with *M. massiliense* and additional signal of *M. intracellulare*.

We conducted further investigations using DNA sequencing to address the discrepancies between the SOC and NeoPlex assay results. Primer sets specific to *M. avium*, *M. intracellulare*, *M. abscessus*, and MTBC were used for sequencing samples with concordant and additional findings. All the isolates were successfully amplified (Figure 2) and accurately identified at a species level. The isolates of *M. avium* and *M. kansasii* initially identified as NTM by the NeoPlex assay were confirmed as *M. avium* and *M. kansasii* using the 16S rRNA and *rpoB* primers.

### 3.3. Sensitivity and Specificity of NeoPlex

The NeoPlex assay demonstrated 100% sensitivity and specificity for detecting *M. intracellulare*, *M. abscessus*, *M. massiliense*, NTM, and MTBC (Table 5); a sensitivity of 90.9% and specificity of 100% for detecting *M. avium*; and a sensitivity of 75.0% and specificity of 100% for detecting *M. kansasii*. The overall sensitivity for the five NTM species was 95.0%, with a specificity of 100%.

## 4. Discussion

Rapid differentiation between MTBC and NTM, as well as species identification of NTM in clinical samples, is crucial for effective patient management, infection control, and the selection of appropriate therapeutic regimens [20]. The findings of this study highlight the effectiveness of the NeoPlex assay for accurately identifying and differentiating between MTBC and the five most common NTM species. To our knowledge, this is the first study to comprehensively evaluate the NeoPlex TB/NTM-5 assay. The NeoPlex assay demonstrated accuracy and reliability and correctly identified 95.6% of isolates grown on liquid or solid culture.

Antimicrobial agents with clinically established breakpoints vary among *M. avium* complex, *M. kansasii*, and *M. abscessus* complex [21]. Accordingly, the treatment guidelines for NTM pulmonary disease differ among these species [4]. Thus, accurate differentiation of these NTM species is important. In this study, the NeoPlex assay detected *M. intracellulare*, *M. abscessus*, and *M. massiliense* with 100% sensitivity and specificity. Although the assay exhibited lower sensitivity for *M. avium* and *M. kansasii* than the aforementioned species, these targets were identified with no false positives (100% specificity), demonstrating that the NeoPlex assay is a reliable tool for distinguishing between clinically common NTM species.

*M. kansasii* is one of the most virulent and clinically significant NTM in human medicine. Molecular typing has revealed that *M. kansasii* comprises at least six distinct subtypes, which differ in prevalence and clinical relevance [22,23]. Recently, based on genome-wide average nucleotide identity, it was proposed that these subtypes should be more accurately designated [24,25]. The six subtypes are now recognized as *M. kansasii* (formerly subtype I), *Mycobacterium persicum* (II), *Mycobacterium pseudokansasii* (III), *Mycobacterium ostraviense* (IV), *Mycobacterium innocens* (V), and *Mycobacterium attenuatum* (VI). Together with *Mycobacterium gastri*, these subtypes form the *M. kansasii* complex [22]. In our study, among eight *M. kansasii* isolates identified by the LPA, two discordant results were observed using the NeoPlex assay. One case involved a multispecies infection with *M. avium*, and the other was a single infection with *M. kansasii* misidentified as NTM. Further sequencing of the isolate misidentified as NTM by the NeoPlex assay revealed it to be *M. attenuatum*. This finding was discussed with GeneMatrix, which conducted in silico analysis and reported that their specific primer design targeted sequences excluding *M. attenuatum*. Although the exact sequence used by the LPA is unknown, a plausible explanation for the NeoPlex assay’s lower sensitivity to *M. kansasii* is that the LPA may employ a universal target sequence to include most, if not all, *M. kansasii* complex members, whereas the NeoPlex assay uses a more specific target. Given the clinical importance of detecting the entire *M. kansasii* complex, it may be beneficial for GeneMatrix to revisit their target sequence design to enhance the NeoPlex assay’s sensitivity for *M. kansasii* complex. For *M. avium*, the NeoPlex assay failed to identify two out of 22 isolates. Similar to the *M. kansasii* cases, this discrepancy may be due to differences in target sequences between the assays. Notably, *M. avium* can be subdivided into four distinct subspecies [26], and genetic variability among these strains may impact the sensitivity of the NeoPlex assay. Further studies are warranted to address these limitations and improve assay performance.

Multispecies NTM infections are not widely recognized, and their clinical implications are only beginning to be investigated. As with single NTM species, the distribution of multispecies NTM infections varies geographically [2]. In Korea, the proportion of multispecies NTM infections ranges from 8.5% to 14.6% of all NTM infections [2,27]. This proportion is comparable to the 7.5% reported in a study conducted in Southwest China [28], but lower than the 30.1% reported in a study conducted in Singapore [29]. The disease status and clinical outcomes of patients with multispecies NTM infections compared with single-species NTM infections warrant further investigation. A study conducted in Korea revealed that the clinical outcomes of NTM coinfections were similar to those of single-species NTM infections [27], whereas a study conducted in Japan revealed that coinfections could lead to more severe disease [30]. These findings highlight the importance of detecting NTM coinfections in patient management and the need for further research. In this study, all isolates from multiple NTM infections were correctly identified using the NeoPlex assay. In addition, the NeoPlex assay detected additional mycobacterial nucleic acids that were not detected by the LPA. These findings suggest that the NeoPlex assay offers enhanced detection sensitivity, which may be critical in cases of coinfection or in which minor species are not detected using traditional methods. The treatment regimen for NTM pulmonary disease varies depending on the causative species. For *M. avium* complex infections, a three-drug regimen consisting of macrolides, ethambutol, and rifampicin is commonly used [4]. However, *M. abscessus* and *M. massiliense* are resistant to ethambutol and rifampicin, necessitating a macrolide-based multidrug regimen combined with intravenous antibiotics during the initial phase. Since clinical characteristics alone cannot differentiate between single- and multispecies infections [27], the ability of the NeoPlex assay to detect multispecies infections could have important clinical relevance. In our study, the NeoPlex assay identified additional *M. avium* complex infections in samples already infected with *M. abscessus* complex. Although this may have represented a minor morphotype in vitro due to the slower growth pattern of *M. avium* complex compared to the rapid growth of *M. abscessus* complex, the in vivo dynamics of multispecies infections could differ. Therefore, patients may have benefited from more tailored therapy if these results were applied, even though clinical guidelines currently lack treatment protocols for multispecies NTM infections [4]. Similarly, the detection of additional *M. intracellulare* in addition to *M. kansasii* could have provided clinical value, as treatment regimens vary between these two NTM species. The incidence, prevalence, clinical features, and optimal treatment strategies for NTM coinfections remain underexplored [27]. Accurate detection of multispecies NTM infections may serve as an essential starting point for future research. Given the capability of the LPA to detect multispecies infections within a single specimen, the detection of additional species using the NeoPlex assay in this study was unexpected. The authors discussed the underperformance of the LPA with staff working in the KIT laboratory. In the KIT laboratory, each specimen from MGIT or Ogawa media was inoculated on Middlebrook 7H11 agar before performing the LPA. Despite observing different colony morphotypes, which are indicative of multispecies infections, technicians typically use the predominant single-colony morphotype for LPA, potentially missing co-infecting species. This approach could explain the detection of additional mycobacterial species by the NeoPlex assay in this study. Possibly, differences in sensitivity between methods is another possible explanation for the detection of additional species using the NeoPlex assay. Therefore, detecting all possible NTM species based on colony morphotypes using LPA or other molecular methods, such as the current assay, is necessary to ensure appropriate patient management.

NTM and TB both cause pulmonary and extrapulmonary disease, share similar clinical presentations, and can be present as coinfections [31]. Previous studies have found that NTM and TB coinfection can occur but is infrequent. A population-based cohort study conducted in Taiwan found that 2.8% of individuals with confirmed TB harbored NTM coinfection [31]. In a national prevalence study conducted in Zambia, 0.2% (13 of 6123 individuals) had confirmed TB/NTM coinfection [32]. A nationwide retrospective multicenter cohort study conducted in Belgium showed that 1.4% (35 of 2569) of individuals with culture-positive pulmonary TB had NTM coinfection [33]. In a study conducted in China, 8% of patients with NTM infections also had bacterial evidence of TB [34]. These findings suggest that TB/NTM coinfection is not uncommon and can hinder the diagnosis. Thus, clinicians should be vigilant regarding TB/NTM coinfection in TB-endemic regions [31]. Accordingly, to detect cases of coinfection and perform careful laboratory procedures to detect multiple species from mycobacterial cultures, conducting nucleic acid amplification tests that simultaneously detect TB and NTM is important [20]. In our study, additional TB nucleic acids were detected using the NeoPlex assay in one specimen with *M. massiliense* infection, which demonstrates its ability to simultaneously detect both TB and NTM. In most clinical microbiology laboratories in Korea, TB differentiation from NTM in positive AFB cultures is performed using the MPT64 antigen test owing to its rapid turnaround time and ease of use. However, previous studies have shown that the MPT64 antigen test has limited sensitivity at detecting *M. tuberculosis* lineages 5 and 6, in which MPT64 protein expression is low [35,36]. Certain BCG strains may also test negative [37]. A recent study showed that isolates containing the 63 bp deletion variant of the *mpt64* gene belonged to Clade A within the *M. tuberculosis* L4.2.2 lineage, indicating that the MPT64 antigen test is also not suitable for detecting this lineage [38]. This indicates that in regions where Clade A isolates are prevalent (such as China or Vietnam), or where lineage 5 and 6 strains are common (e.g., West Africa), as well as in cases in which BCG infection is suspected (e.g., patients with a history of bladder BCG therapy), MPT64-negative test results should be verified using alternative identification methods before classifying them as NTM.

The detection of additional TB infection in a specimen coinfected with *M. massiliense* in our study warrants further discussion. Hidden TB infections may occur in patients co-infected with rapidly growing NTM, as demonstrated in this case. Clinicoradiologic findings of the patient suggested TB-NTM coinfection, with radiologic evidence of TB reactivation in an old scar and bronchiectasis, a condition strongly associated with NTM infections [39]. Owing to the differential growth rates of the rapidly growing *M. massiliense* and the slowly growing MTBC, *M. massiliense* was more predominant, as reflected by the Ct values. Unfortunately, the patient’s transfer to another hospital precluded further assessment of clinical outcomes. However, the clinical presentation and PCR results were consistent with TB-NTM coinfection. The NeoPlex assay showed significant potential for identifying hidden TB infections in specimens in which rapidly growing NTM are predominant, particularly when MPT64 antigen test results are negative. Its implementation could enhance diagnostic accuracy in complex clinical scenarios.

Although the NeoPlex assay has several advantages, including reduced turnaround time and a user-friendly design, it is important to consider the limitations inherent to multiplex platforms. Although five of the most prevalent NTM species were successfully detected, the assay does not detect some other less common but clinically significant NTM species, such as *Mycobacterium chelonae*, *Mycobacterium malmoense*, and *Mycobacterium fortuitum* complex. Consequently, the NeoPlex assay should not replace established methods for NTM species identification, such as DNA sequencing, LPA, or MALDI-TOF mass spectrometry. Instead, the assay can be used in conjunction with these methods, depending on the laboratory capabilities. Alternatively, it may serve as a rapid screening tool for the five most common NTM species and MTBC following a positive AFB culture. Specimens identified as NTM without the five NTM signals could then be referred to specialized facilities that offer more comprehensive NTM species identification. Future research should explore the cost-effectiveness of implementing the NeoPlex assay in different healthcare settings, particularly in low-resource environments, in which rapid and accurate diagnosis is essential for effective TB control and management of NTM infections.

This study had several limitations. First, the relatively small sample size from a single center may account for the low sensitivity observed for *M. kansasii*. Second, the study did not evaluate simultaneous head-to-head comparisons with SOC testing, thereby preventing a comparison of the turnaround time between methods. Similarly, our analysis mainly compared the diagnostic accuracy of the NeoPlex assay with the LPA. Future studies are necessary to assess the performance of the NeoPlex assay in comparison to other commercial TB/NTM detection kits or sequencing methods, which could enhance our understanding of its diagnostic capabilities. Third, this study only used samples that were positive on culture, leading to possible sampling bias. Direct detection of nucleic acids from raw specimens, such as pulmonary samples (e.g., sputum or bronchoalveolar lavage) or extrapulmonary samples (e.g., tissue or pus), could have a greater impact. Factors such as prior antibiotic use, host-produced antimicrobial peptides, the fastidious nature of AFB, low bacterial titers, and potential contamination from other bacteria may limit the recovery of AFB in culture. Therefore, future investigations should focus on assessing the performance of the NeoPlex assay directly on unprocessed specimens to address these limitations. Such research may enhance the speed of NTM species identification and have important implications for clinical practice.

## 5. Conclusions

The NeoPlex TB/NTM-5 assay demonstrated high concordance with the standard diagnostic methods for detecting MTBC and NTM, as well as differentiating five common NTM species: *M. intracellulare*, *M. avium*, *M. kansasii*, *M. abscessus*, and *M. massiliense*. Notably, the assay identified additional NTM species that were undetected by the LPA. Furthermore, it demonstrated significant potential in uncovering hidden MTBC infections in specimen coinfected with rapidly growing NTM species. These findings underscore its value in improving diagnostic accuracy and patient care. Future studies incorporating clinical data and testing with raw specimens are warranted to further validate these results.

## Figures and Tables

**Table 1:** Clinical strains tested in this study (*n* = 91).

*Mycobacterial* Species	No. of Isolates
Nontuberculous mycobacteria	55
Single species	38
*M. avium* complex	
*M. intracellulare*	10
*M. avium*	8
*M. kansasii*	7
*M. abscessus* complex	
*M. abscessus*	8
*M. massiliense*	5
Multispecies	17
*M. intracellulare* and *M. avium*	7
*M. intracellulare* and *M. abscessus*	4
*M. intracellulare* and *M. massiliense*	1
*M. intracellulare* and *M. gordonae*	1
*M. avium* and *M. kansasii*	1
*M. avium* and *M. massiliense*	1
*M. abscessus* and *M. fortuitum* complex	1
*M. intracellulare, M. avium* and *M. abscessus*	1
*M. tuberculosis* complex	36

**Table 2:** Summary of targets and cut-off values of the NeoPlex TB/NTM detection kit.

Target	Fluorescence Dye	Primer Target	Probe	Cut Off	Melting Temperature (°C)
*Mycobacterium tuberculosis* complex	FAM	IS*6110*	Taqman	<40 (Ct)	NA
*Mycobacterium* species	Cal Red 610	16S rRNA	Taqman	<40 (Ct)	NA
Internal control	HEX	*UBB*	Taqman	<40 (Ct)	NA
*Mycobacterium intracellulare*	Cal Red 610	Proprietary	C-Tag	≥74.9 (-dRFU/dT)	71 ± 2
*Mycobacterium avium*	HEX	Proprietary	C-Tag	≥75.0 (-dRFU/dT)	73 ± 2
*Mycobacterium kansasii*	Quasar 670	Proprietary	C-Tag	≥75.4 (-dRFU/dT)	72 ± 2
*Mycobacterium abscessus*	FAM	Proprietary	C-Tag	≥74.7 (-dRFU/dT)	63 ± 2
*Mycobacterium massiliense*	HEX	Proprietary	C-Tag	≥74.8 (-dRFU/dT)	64 ± 2

Ct, cycle threshold; dRFU, order derivative relative fluorescence unit; dT, time derivative; FAM, fluorescein amidite; HEX, hexachlorofluorescein; NA, not applicable; NTM, nontuberculous mycobacteria; TB, tuberculosis.

**Table 3:** Comparison of the results of testing using the NeoPlex TB/NTM-5 assay and standard of care testing using the line-probe assay.

Standard of Care Testing	NeoPlex TB/NTM-5 Assay	No.
Concordant results (*n* = 79)		
*M. intracellulare*	*M. intracellulare*	9
*M. avium*	*M. avium*	6
*M. kansasii*	*M. kansasii*	5
*M. abscessus*	*M. abscessus*	5
*M. massiliense*	*M. massiliense*	3
*M. intracellulare* and *M. avium*	*M. intracellulare*/*M. avium*	6
*M. intracellulare* and *M. abscessus*	*M. intracellulare/M. abscessus*	4
*M. intracellulare* and *M. massiliense*	*M. intracellulare/M. massiliense*	1
*M. intracellulare* and *M. gordonae*	*M. intracellulare*	1
*M. avium* and *M. massiliense*	*M. avium/M. massiliense*	1
*M. abscessus* and *M. fortuitum* complex	*M. abscessus*	1
*M. intracellulare, M. avium* and *M. abscessus*	*M. intracellulare/M. avium/M. abscessus*	1
*M. tuberculosis* complex	*M. tuberculosis* complex	36
Concordant results plus additional results detected using the NeoPlex assay (*n* = 8)		
*M. intracellulare*	*M. intracellulare/M. avium*	1
*M. avium*	*M. avium/M. intracellulare*	1
*M. kansasii*	*M. kansasii/M. intracellulare*	1
*M. abscessus*	*M. abscessus/M. avium/M. intracellulare*	2
*M. abscessus*	*M. abscessus/M. avium*	1
*M. massiliense*	*M. massiliense/M. intracellulare*	1
*M. massiliense*	*M. massiliense/M. tuberculosis* complex	1
Partial identification results using the NeoPlex assay (*n* = 2)		
*M. intracellulare* and *M. avium*	*M. intracellulare*	1
*M. avium* and *M. kansasii*	*M. avium*	1
Misidentification results using the NeoPlex assay (*n* = 2)		
*M. kansasii*	NTM	1
*M. avium*	NTM	1

NTM, nontuberculous mycobacteria; TB, tuberculosis.

**Table 4:** Summary of isolates with concordant plus additional, partial, and misidentification results detected using the NeoPlex TB/NTM-5 assay.

			Performance of NeoPlex TB/NTM-5 Assay				
Study Number	Standard of Care Testing	Culture Used	MTBC Ct	*Mycobacterium* spp. Ct	IC Ct	TB/NTM Result	Melting Curve Analysis
Concordant plus additional results detected using the NeoPlex assay							
2	*M. abscessus*	MGIT	NA	20.18	37.5	NTM	*M. abscessus/M. avium/M. intracellulare*
5	*M. abscessus*	MGIT	NA	25.08	NA	NTM	*M. abscessus/M. avium*
7	*M. abscessus*	MGIT	NA	19.50	38.71	NTM	*M. abscessus/M. avium/M. intracellulare*
8	*M. massiliense*	Ogawa	39.20	20.17	NA	TB and NTM	*M. massiliense*
15	*M. intracellulare*	MGIT	NA	27.25	NA	NTM	*M. intracellulare/M. avium*
18	*M. avium*	Ogawa	NA	26.21	NA	NTM	*M. avium/M. intracellulare*
30	*M. kansasii*	MGIT	NA	23.56	NA	NTM	*M. kansasii/M. intracellulare*
33	*M. massiliense*	MGIT	NA	22.23	NA	NTM	*M. massiliense/M. intracellulare*
Partial identification results using the NeoPlex assay							
40	*M. intracellulare* and *M. avium*	MGIT	NA	24.66	NA	NTM	*M. intracellulare*
48	*M. avium* and *M. kansasii*	MGIT	NA	25	NA	NTM	*M. avium*
Misidentification results using the NeoPlex assay							
9	*M. kansasii*	Ogawa	NA	20.90	NA	NTM	Not detected
26	*M. avium*	Ogawa	NA	25.58	NA	NTM	Not detected

Ct, cycle threshold; IC, internal control; MGIT, mycobacterial growth indicator tube; MTBC, *Mycobacterium tuberculosis* complex; NA, not applicable; NTM, nontuberculous mycobacteria.

**Table 5:** Sensitivity and specificity of the NeoPlex TB/NTM-5 assay for detecting different targets.

NeoPlex TB/NTM-5 Target	TP	TN	FN	FP	Sensitivity (95% CI)	Specificity (95% CI)
*Mycobacterium intracellulare*	29	62	0	0	100% (88.3–100%)	100% (94.2–100%)
*Mycobacterium avium*	20	69	2	0	90.9% (72.2–98.4%)	100% (94.7–100%)
*Mycobacterium kansasii*	6	83	2	0	75.0% (40.9–95.6%)	100% (95.6–100%)
*Mycobacterium abscessus*	14	77	0	0	100% (78.5–100%)	100% (95.3–100%)
*Mycobacterium massiliense*	7	84	0	0	100% (64.6–100%)	100% (95.6–100%)
All five species	76	375	4	0	95.0% (87.8–98.0%)	100% (99.0–100%)
NTM	55	36	0	0	100% (93.5–100%)	100% (90.4–100%)
MTBC	37	54	0	0	100% (90.6–100%)	100% (93.4–100%)
All NTM and MTBC	92	90	0	0	100% (96.0–100%)	100% (95.9–100%)

CI, confidence interval; FN, false negative; FP, false positive; MTBC, *Mycobacterium tuberculosis* complex; NTM, nontuberculous mycobacteria; TB, tuberculosis; TN, true negative; TP, true positive.

## Data Availability

All data pertinent to the study are included in the article or as Supplementary Information.

## Supplementary Materials

The following supporting information can be downloaded at https://www.mdpi.com/article/10.3390/microorganisms13010026/s1, Figure S1. Examples of melting curve plots for different NTM species; Table S1. Interpretation criteria according to the cycle threshold (Ct) value and melting curve analysis; Table S2. Line listing of the results of NeoPlex TB/NTM-5 assay for each of the 91 tested specimens.
