# Supplementary material for: Evaluation of a Commercial Multiplex Real-Time PCR with Melting Curve Analysis for the Detection of Mycobacterium tuberculosis Complex and Five Nontuberculous Mycobacterial Species
Source: Microorganisms. 2024 Dec 26;13(1):26. doi: 10.3390/microorganisms13010026 (PMC11767457; doi:10.3390/microorganisms13010026)
Supplement: Supplementary file 1 [file microorganisms-13-00026-s001.zip › microorganisms-3384315-supplementary.pdf]

Supplementary Materials

Evaluation of a Commercial Multiplex Real-Time PCR with Melting Curve Analysis for the Detection of *Mycobacterium tuberculosis* Complex and Five Nontuberculous Mycobacterial Species

List of Supplementary Materials

Supplementary Figures

Figure S1. Examples of melting curve plots for different NTM species.

Supplementary Tables

Table S1. Interpretation criteria according to the cycle threshold (Ct) value and melting curve analysis.

Table S2. Line listing of the results of NeoPlex TB/NTM-5 assay for each of the 91 tested specimens.

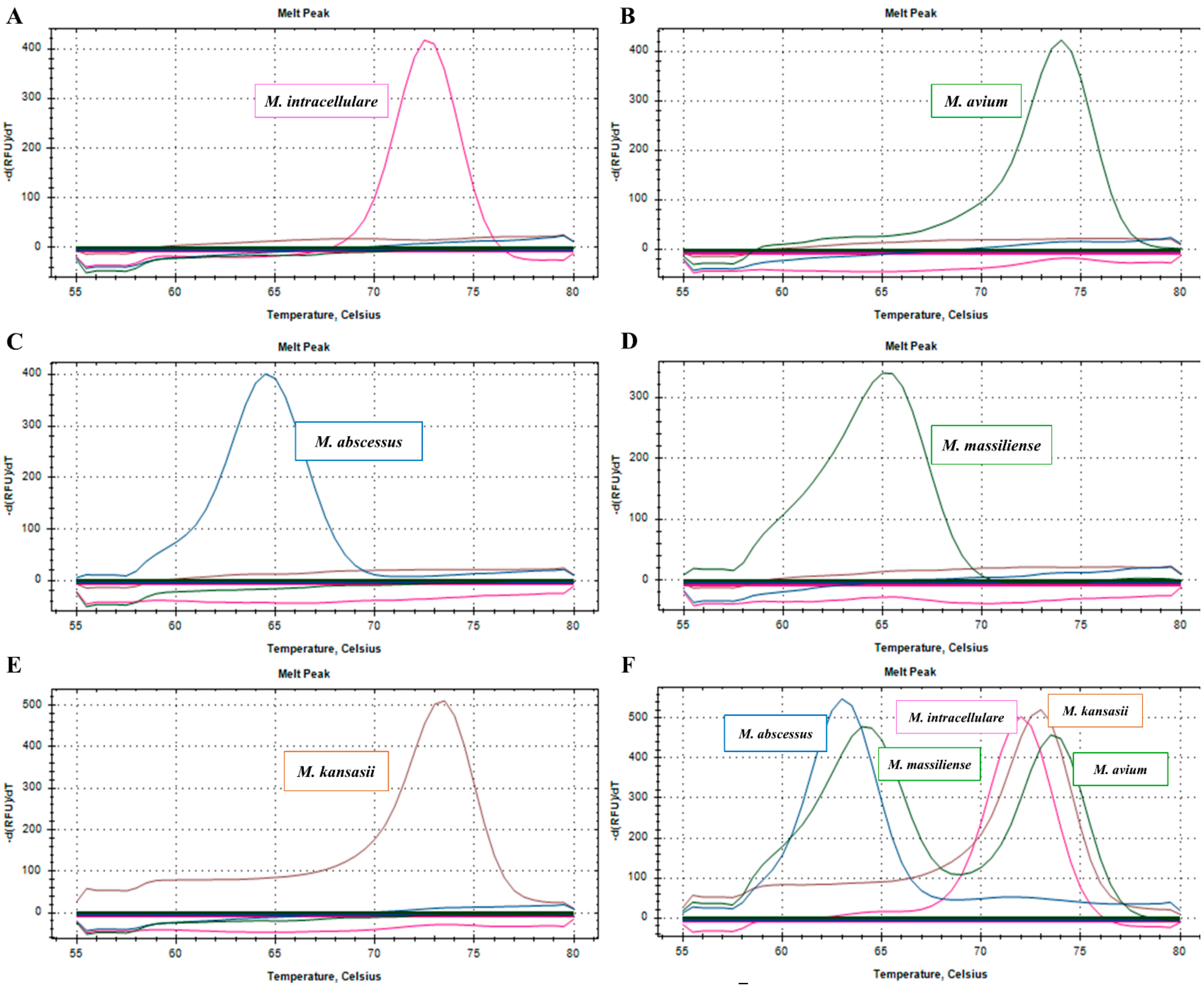
A
Melt Peak
M. intracellulare
-d(RFU)/dT
Temperature, Celsius
B
Melt Peak
M. avium
-d(RFU)/dT
Temperature, Celsius
C
Melt Peak
M. abscessus
-d(RFU)/dT
Temperature, Celsius
D
Melt Peak
M. massiliense
-d(RFU)/dT
Temperature, Celsius
E
Melt Peak
M. kansasii
-d(RFU)/dT
Temperature, Celsius
F
Melt Peak
M. abscessus
M. intracellulare
M. kansasii
M. massiliense
M. avium
-d(RFU)/dT
Temperature, Celsius

Figure S1. Examples of melting curve plots for different NTM species. (A) Detection of *M. intracellulare* using Cal Red 610 fluorescent dye. (B) Detection of *M. avium* using hexachlorofluorescein (HEX) fluorescent dye. (C) Detection of *M. abscessus* using FAM fluorescent dye. (D) Detection of *M. massiliense* using HEX fluorescent dye. (E) Detection of *M. kansasii* using Quasar 670 fluorescent dye. (F) All five melting curves for the five NTM species superimposed for comparison. FAM, fluorescein amidite; HEX, hexachlorofluorescein.

Table S1. Interpretation criteria according to the cycle threshold (Ct) value and melting curve analysis.

	Ct value positivity of real-time PCR			Florescence positivity of melting curve analysis					
Case scenario[a]	FAM (MTBC)	Cal Red 610 (*Mycobacterium* spp.)	HEX (IC)	MIT	MAV	MKS	MAB	MMS	Interpretation
1	+	−	+/−	−	−	−	−	−	MTBC
2	+	+	+/−	−	−	−	−	−	MTBC or MTBC/NTM co-infection[b]
3	+	+	+/−	+	−	−	−	−	MTBC/NTM (MIT) co-infection
4	−	+	+/−	−	−	−	−	−	NTM[c]
5	−	+	+/−	+	−	−	−	−	NTM (MIT)
6	−	+	+/−	−	+	−	−	−	NTM (MAV)
7	−	+	+/−	−	−	+	−	−	NTM (MKS)
8	−	+	+/−	−	−	−	+	−	NTM (MAB)
9	−	+	+/-	−	−	−	−	+	NTM (MMS)
10	−	+	+/-	+	−	−	−	+	NTM (MIT, and MMS)
11	−	+	+/-	+	−	+	−	+	NTM (MIT, MKS, and MMS)
11	−	−	+	−	−	−	−	−	Negative
12	−	−	-	−	−	−	−	−	Retest

Ct, cycle threshold; FAM, fluorescein amidite; HEX, hexachlorofluorescein; IC, internal control; MAB, *Mycobacterium abscessus;* MAV, *Mycobacterium avium;* MIT, *Mycobacterium intracellulare*; MKS, *Mycobacterium kansasii;* MMS*, Mycobacterium massiliense*; MTBC, *Mycobacterium tuberculosis* complex.

[a]Not every possible scenario is included; other clinical scenarios are also possible (i.e., NTM (MIT, MAV, and MAB)).

[b]FAM Ct value < Cal Red 610 Ct value: MTBC infection only; FAM Ct value ≥ Cal Red 610 Ct value: MTBC and NTM co-infection.

[c]NTM species other than the five target species are suspected.

Table S2. Line listing of the results of NeoPlex TB/NTM-5 assay for each of the 91 tested specimens.

		Performance of NeoPlex TB/NTM-5					
Study number	Reference	Culture used	TB Ct value	*Mycobacterium* spp. Ct value	IC Ct value	TB/NTM result	Melting curve analysis
1	*M. abscessus*	MGIT	NA	20.39	NA	NTM	*M. abscessus*
2	*M. abscessus*	MGIT	NA	20.18	37.5	NTM	*M. abscessus, M. avium,* and *M. intracellulare*
3	*M. massiliense*	Ogawa	NA	18.67	NA	NTM	*M. massiliense*
4	*M. abscessus*	Ogawa	NA	22.59	NA	NTM	*M. abscessus*
5	*M. abscessus*	MGIT	NA	25.08	NA	NTM	*M. abscessus* and *M. avium*
6	*M. massiliense*	Ogawa	NA	25.96	NA	NTM	*M. massiliense*
7	*M. abscessus*	MGIT	NA	19.50	38.71	NTM	*M. abscessus, M. avium,* and *M. intracellulare*
8	*M. massiliense*	Ogawa	39.20	20.17	NA	TB and NTM	*M. massiliense*
9	*M. kansasii*	Ogawa	NA	20.90	NA	NTM	Not detected
10	*M. massiliense*	Ogawa	NA	24.32	NA	NTM	*M. massiliense*
11	*M. intracellulare*	MGIT	NA	27.76	NA	NTM	*M. intracellulare*
12	*M. intracellulare*	MGIT	NA	26.42	NA	NTM	*M. intracellulare*
13	*M. intracellulare*	MGIT	NA	22.99	NA	NTM	*M. intracellulare*
14	*M. intracellulare*	MGIT	NA	26.50	NA	NTM	*M. intracellulare*
15	*M. intracellulare*	MGIT	NA	27.25	NA	NTM	*M. intracellulare* and *M. avium*
16	*M. intracellulare*	Ogawa	NA	27.48	NA	NTM	*M. intracellulare*
17	*M. intracellulare*	MGIT	NA	27.38	NA	NTM	*M. intracellulare*
18	*M. avium*	Ogawa	NA	26.21	NA	NTM	*M. avium* and *M. intracellulare*
19	*M. intracellulare*	Ogawa	NA	25.21	NA	NTM	*M. intracellulare*
20	*M. avium*	MGIT	NA	27.50	NA	NTM	*M. avium*
21	*M. avium*	Ogawa	NA	25.15	NA	NTM	*M. avium*
22	*M. avium*	Ogawa	NA	27.63	NA	NTM	*M. avium*
23	*M. intracellulare*	MGIT	NA	24.48	38.68	NTM	*M. intracellulare*
24	*M. avium*	Ogawa	NA	23.45	NA	NTM	*M. avium*
25	*M. avium*	MGIT	NA	21.84	34.18	NTM	*M. avium*
26	*M. avium*	Ogawa	NA	25.58	NA	NTM	Not detected
27	*M. kansasii*	MGIT	NA	21.20	NA	NTM	*M. kansasii*
28	*M. abscessus*	MGIT	NA	23.85	NA	NTM	*M. abscessus*
29	*M. abscessus*	MGIT	NA	19.53	NA	NTM	*M. abscessus*
30	*M. kansasii*	MGIT	NA	23.56	NA	NTM	*M. kansasii* and *M. intracellulare*
31	*M. kansasii*	MGIT	NA	19.05	39.46	NTM	*M. kansasii*

Study number	Reference	Performance of NeoPlex TB/NTM-5					
		Culture used	TB Ct value	*Mycobacterium* spp. Ct value	IC Ct value	TB/NTM result	Melting curve analysis
32	*M. abscessus*	MGIT	NA	18.03	38.21	NTM	*M. abscessus*
33	*M. massiliense*	MGIT	NA	22.23	NA	NTM	*M. massiliense* and *M. intracellulare*
34	*M. kansasii*	MGIT	NA	28.52	NA	NTM	*M. kansasii*
35	*M. kansasii*	MGIT	NA	21.09	NA	NTM	*M. kansasii*
36	*M. kansasii*	MGIT	NA	20.87	NA	NTM	*M. kansasii*
37	*M. intracellulare* and *M. abscessus*	MGIT	NA	21.39	38.07	NTM	*M. intracellulare* and *M. abscessus*
38	*M. intracellulare* and *M. massiliense*	Ogawa	NA	22.68	NA	NTM	*M. intracellulare* and *M. massiliense*
39	*M. intracellulare* and *M. avium*	MGIT	NA	26.35	NA	NTM	*M. intracellulare* and *M. avium*
40	*M. intracellulare* and *M. avium*	MGIT	NA	24.66	NA	NTM	*M. intracellulare*
41	*M. intracellulare* and *M. avium*	MGIT	NA	21.34	31.21	NTM	*M. intracellulare* and *M. avium*
42	*M. intracellulare* and *M. abscessus*	Ogawa	NA	21.35	NA	NTM	*M. intracellulare* and *M. abscessus*
43	*M. intracellulare* and *M. avium*	MGIT	NA	25.49	NA	NTM	*M. intracellulare* and *M. avium*
44	*M. intracellulare, M. avium* and *M. abscessus*	MGIT	NA	19.81	39.32	NTM	*M. intracellulare, M. avium,* and *M. abscessus*
45	*M. intracellulare* and *M. abscessus*	MGIT	NA	24.91	39.76	NTM	*M. intracellulare* and *M. abscessus*
46	*M. intracellulare* and *M. abscessus*	MGIT	NA	25.27	NA	NTM	*M. intracellulare* and *M. abscessus*
47	*M. intracellulare* and *M. avium*	MGIT	NA	25	NA	NTM	*M. intracellulare* and *M. avium*
48	*M. avium* and *M. kansasii*	MGIT	NA	26.17	34.22	NTM	*M. avium*
49	*M. avium* and *M. massiliense*	MGIT	NA	26.27	NA	NTM	*M. avium* and *M. massiliense*
50	*M. intracellulare* and *M. avium*	MGIT	NA	27.6	NA	NTM	*M. intracellulare* and *M. avium*
51	*M. intracellulare* and *M. avium*	MGIT	NA	22.94	NA	NTM	*M. intracellulare* and *M. avium*

Study number	Reference	Performance of NeoPlex TB/NTM-5					
		Culture used	TB Ct value	*Mycobacterium* spp. Ct value	IC Ct value	TB/NTM result	Melting curve analysis
52	*M. intracellulare* and *M. gordonae*	MGIT	NA	22.22	NA	NTM	*M. intracellulare*
53	*M. intracellulare*	MGIT	NA	23.37	NA	NTM	*M. intracellulare*
54	*M. abscessus* and *M. fortuitum* complex	MGIT	NA	28.99	39.77	NTM	*M. abscessus*
55	*M. avium*	Ogawa	NA	29.6	NA	NTM	*M. avium*
56	*M. tuberculosis* complex	MGIT	19.5	27.18	NA	TB	Not detected
57	*M. tuberculosis* complex	MGIT	17.14	23.67	33.41	TB	Not detected
58	*M. tuberculosis* complex	MGIT	18.92	26.09	37.17	TB	Not detected
59	*M. tuberculosis* complex	MGIT	20.6	26.67	39.26	TB	Not detected
60	*M. tuberculosis* complex	MGIT	16.06	22.79	NA	TB	Not detected
61	*M. tuberculosis* complex	MGIT	20.82	28.34	NA	TB	Not detected
62	*M. tuberculosis* complex	MGIT	21.23	28.04	33.23	TB	Not detected
63	*M. tuberculosis* complex	MGIT	15.04	21.56	35.65	TB	Not detected
64	*M. tuberculosis* complex	MGIT	17.21	23.84	35.89	TB	Not detected
65	*M. tuberculosis* complex	MGIT	17.67	24.29	33.73	TB	Not detected
66	*M. tuberculosis* complex	MGIT	18.07	24.25	39.27	TB	Not detected
67	*M. tuberculosis* complex	MGIT	19.92	26.68	38.82	TB	Not detected
68	*M. tuberculosis* complex	MGIT	18.36	24.67	36.06	TB	Not detected
69	*M. tuberculosis* complex	MGIT	15.79	23.51	34.96	TB	Not detected
70	*M. tuberculosis* complex	MGIT	18.36	24.21	NA	TB	Not detected
71	*M. tuberculosis* complex	MGIT	16.21	22.59	35.32	TB	Not detected
72	*M. tuberculosis* complex	MGIT	21.1	28.08	39.2	TB	Not detected
73	*M. tuberculosis* complex	MGIT	18.21	24.28	NA	TB	Not detected
74	*M. tuberculosis* complex	Ogawa	17.78	23.78	NA	TB	Not detected
75	*M. tuberculosis* complex	Ogawa	18.77	24.2	NA	TB	Not detected
76	*M. tuberculosis* complex	Ogawa	19.83	25.84	NA	TB	Not detected
77	*M. tuberculosis* complex	Ogawa	21.98	27.92	NA	TB	Not detected
78	*M. tuberculosis* complex	Ogawa	15.55	22.38	NA	TB	Not detected
79	*M. tuberculosis* complex	Ogawa	13.34	19.93	39.17	TB	Not detected
80	*M. tuberculosis* complex	Ogawa	19.9	26.36	NA	TB	Not detected
81	*M. tuberculosis* complex	Ogawa	18.3	24.79	NA	TB	Not detected
82	*M. tuberculosis* complex	Ogawa	15.53	20.64	NA	TB	Not detected
83	*M. tuberculosis* complex	Ogawa	17.39	23.42	NA	TB	Not detected
84	*M. tuberculosis* complex	Ogawa	22.8	29.15	NA	TB	Not detected

Study number	Reference	Performance of NeoPlex TB/NTM-5					
		Culture used	TB Ct value	*Mycobacterium* spp. Ct value	IC Ct value	TB/NTM result	Melting curve analysis
85	*M. tuberculosis* complex	Ogawa	15.81	21.87	NA	TB	Not detected
86	*M. tuberculosis* complex	Ogawa	13.45	19	NA	TB	Not detected
87	*M. tuberculosis* complex	Ogawa	18.14	24.39	NA	TB	Not detected
88	*M. tuberculosis* complex	Ogawa	20.49	27.19	39.56	TB	Not detected
89	*M. tuberculosis* complex	Ogawa	18.68	25.22	NA	TB	Not detected
90	*M. tuberculosis* complex	Ogawa	14.74	20.57	NA	TB	Not detected
91	*M. tuberculosis* complex	Ogawa	14.9	21.03	NA	TB	Not detected

Ct, cycle threshold; IC, internal control; MGIT, Mycobacteria Growth Indicator Tube; NA, not applicable; NTM, nontuberculous mycobacteria; TB, tuberculosis.
